# Influence of coronary architecture on the variability in myocardial infarction induced by coronary ligation in rats

**DOI:** 10.1371/journal.pone.0183323

**Published:** 2017-08-24

**Authors:** Satoshi Kainuma, Shigeru Miyagawa, Satsuki Fukushima, Hirotsugu Tsuchimochi, Takashi Sonobe, Yutaka Fujii, James T. Pearson, Atsuhiro Saito, Akima Harada, Koichi Toda, Mikiyasu Shirai, Yoshiki Sawa

**Affiliations:** 1 Department of Cardiovascular Surgery, Osaka University Graduate School of Medicine, Suita, Osaka, Japan; 2 Department of Cardiac Physiology, National Cerebral and Cardiovascular Center Research Institute, Suita, Osaka, Japan; 3 Department of Physiology, Monash University, Clayton, Australia; Universidad de Buenos Aires, ARGENTINA

## Abstract

It has been shown that the size of myocardial infarction in rats created by coronary ligation technique is not uniform, varying from 4% to 65%. We hypothesized that infarct size variability induced by coronary artery ligation might be caused by coronary artery branching pattern. Coronary artery angiography was performed in 50 normal Lewis rats and in chronic myocardial infarction models in which coronary artery was ligated immediately below the left atrial appendage or 2mm distal to the left atrial appendage (n = 25 for each), followed by histological analysis. Unlike the human, the rats had a single major septal artery arising from the proximal part of the left coronary artery (n = 30) or right coronary artery (n = 20). There were three branching patterns of left circumflex artery (LCX): 33 (66%) had LCX branching peripherally from a long left main coronary artery (LMCA), while the remainder 17 (34%) had the LCX branching from the proximal part of the septal artery or a short LMCA. The rats with distal coronary ligation presented myocardial infarction localized to an anterior territory irrespective of LCX branching pattern. In the rats with proximal coronary ligation, 64% (n = 16) had broad myocardial infarction involving the anterior and lateral territories, while the remainder (36%, n = 9) had myocardial infarction localized to an anterior territory with the intact LCX arising proximally from a short LMCA. The interventricular septum was spared from infarction in all rats because of its anatomical location. Infarct size variations were caused not only by ligation site but also by varying LCX branching patterns. There are potential risks to create different sizes of myocardial infarction, particularly when targeting a broad range of myocardial infarction. The territory of the septal artery always appears to be spared from myocardial infarction induced by the coronary ligation technique.

## Introduction

Rat myocardial infarction models have been widely used, not only for studying the pathophysiology of post-infarct remodeling process, but also for testing the efficacy of pharmacological and/or therapeutic strategies for adverse sequelae [1–3]. Among a variety of surgical manipulations used during the past decade to induce the ischemic event, ligation of the left coronary artery is still the most commonly practiced method [4–6]. In order to verify the impact of different therapeutic treatments and/or interventions on subsequent outcomes in experimental myocardial infarction models, it is critically important to standardize the size and location of myocardial infarction at the time of randomization. However, it is reported that the size of myocardial infarction induced by the coronary ligation technique is not uniform, varying from 4% to 65% in rat models [7]. This varying size of myocardial infarction inherent in the coronary ligation model is attributed at least partly to being unable to occlude the coronary artery exactly at the same point in all animals as the left coronary artery is usually intramyocardial in its proximal region, returning to the surface epicardially at approximately 3 to 4 mm from its origin [8–11]. Another likely factor that contributes to the variability in infarct size may be associated with morphological differences in the pattern of distribution of the coronary artery vessels that are affected by the ligation [12]. We speculate that infarct size variability may be caused not only by the location of the ligation site but also by anatomic variants of coronary branching patterns in different strains of rats. Herein, we aimed to characterize the distribution of the coronary arteries in a rat strain that is routinely used in studies of regenerative therapies and investigated the factors involved in the varying size and location of myocardial infarction that is created by coronary artery ligation.

## Materials and methods

Animal care complied with the “Guide for the Care and Use of Laboratory Animals” (National Institutes of Health publication). Experimental protocols were approved by the Ethics Review Committee for Animal Experimentation of Osaka University Graduate School of Medicine.

### Creation of chronic myocardial infarction rat models

**Fig 1:**
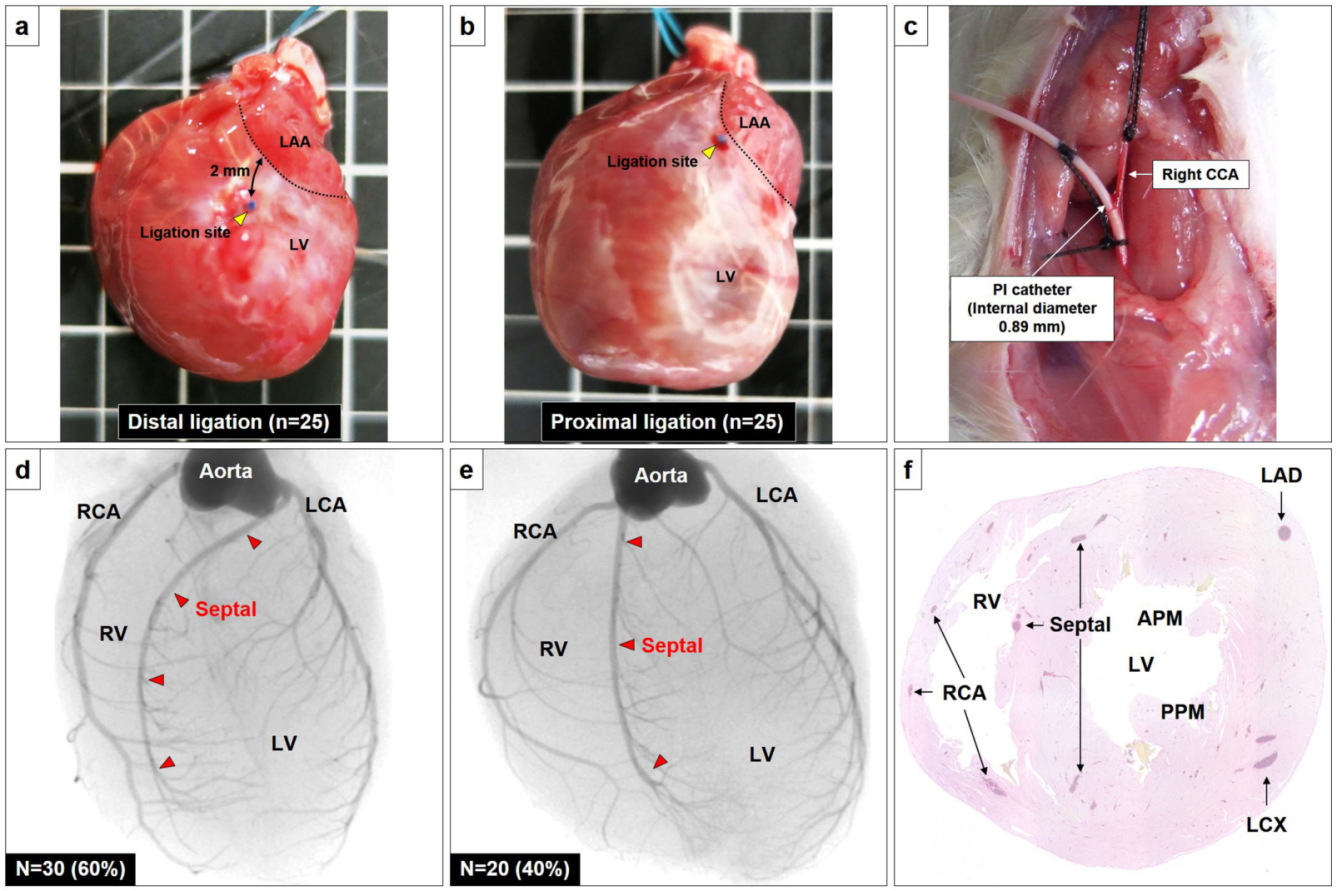
The left coronary artery was permanently ligated 2 mm distal to **(A)** or immediately below **(B)** the LAA. A catheter with an internal diameter of 0.89 mm was inserted from the right carotid artery into the aortic root **(C)**. The septal artery invariably branched off either from proximal part of the LCA (n = 30, 60%) **(D)** or RCA (n = 20, 40%) **(E)**. Representative histological images of left and right ventricular cavities at the level of the papillary muscles in normal rats **(F)**. Abbreviations: LV = left ventricle, LAA = left atrial appendage, CCA = carotid coronary artery, PI = peripherally inserted, LCA = left coronary artery, RCA = right coronary artery, RV = right ventricle, LAD = left anterior descending artery, LCX = left circumflex artery, APM = anterior papillary muscle, PPM = posterior papillary muscle.

Eight-week old female *Lewis rats* (180–200 g, Charles River) were anesthetized with ketamine and xylazine. Under mechanical ventilation (model SN-480-7 Shinano, Tokyo, Japan), a left thoracotomy was performed through the fourth intercostal space, and the lungs were retracted to expose the heart. After the pericardium was opened, the coronary artery was permanently ligated immediately below or 2 mm distal to left atrial appendage with a 7–0 polypropylene suture (Ethicon, Inc., Somerville, N.J.) (Fig 1A and 1B). The ligation was deemed successful when the anterior wall of the LV turned pale.

### Echocardiographic assessment of cardiac function

Cardiac function was evaluated by transthoracic echocardiography at 3 weeks after coronary artery ligation, just before the angiography analysis, using a SONOS 5500 (Philips, Andover, MA, USA) equipped with a 12-MHz annular array transducer under general anaesthesia induced and maintained by inhalation of isoflurane (2%, 0.2 mL/min Mylan; Pittsburgh, PA, USA). The hearts were imaged in short-axis 2D views at the level of the papillary muscles, and the LV end-systolic and end-diastolic dimensions were determined. LV ejection fraction was calculated by Pombo’s method [13].

### Coronary artery angiography

Rats were anesthetized with sodium pentobarbital (60 mg/kg ip) and a catheter with an internal diameter of 0.89 mm (COVIDIEN Ltd, Tokyo, Japan) was inserted from the right carotid artery into the aortic root, followed by systemic heparinization (1000 IU heparin) (Fig 1C). The rats were euthanized with an intraperitoneal injection of pentobarbital (300 mg/kg), then approximately 0.5 mL of a solution consisting of 70% weight/volume barium sulfate (Barytogen, FUSHIMI Pharmaceutical Co, Ltd., Kagawa, Japan) suspended in 7–8% gelatin (GELATIN LEAF 300, YASU CHEMICAL INC., Shiga, Japan) was then injected in a retrograde manner via the catheter using a programmed syringe pump. The heart was immediately harvested and immersed in ice to solidify the contrast agent. Microangiography was performed with an angiography system (MFX-80HK, Hitex Co, Ltd., Osaka, Japan) consisting of an open-type 1 μm microfocus X ray source (L9191, Hamamatsu Photonics Co, Ltd., Hamamatsu, Japan) and a 50/100 mm (2”/4”) dual mode X-ray image intensifier (E5877JCD1-2N, Toshiba Co, Ltd., Tokyo, Japan) set at 60 kV and 60 μA. Using this system, the heart samples could be observed at any desired geometrical magnification and position/orientation.

### Estimation of myocardial infarction size

The heart was cut transversely at the level of papillary muscle and snap-frozen embedded in optimal cutting compound (Tissue-Tek, Sakura Finetek Japan Co, Ltd., Tokyo, Japan) and stored at -80 degrees Celsius. Sections were stained with 0.1% picrosirius red (Sigma Aldrich) to assess myocardial fibrosis in the infarct region. The size of myocardial infarction was calculated as a ratio of the internal perimeter of the infarcted area in relation to the total perimeter of the LV cavity at the level of papillary muscle. The images were examined by optical microscopy (Olympus, Tokyo, Japan) and quantitative morphometric analysis for each sample was performed using Metamorph software (Molecular Devices, Sunnyvale, California, US).

### Statistical analysis

Continuous variables are summarized as means with standard deviations and were compared using an unpaired t-test. Correlations between variables were tested with Pearson’s correlation coefficient (r). All probability values are 2-sided, and values of p<0.05 were considered to indicate statistical significance. Statistical analyses were performed using JMP 7.0 (SAS Institute, Cary, North Carolina).

## Results

### Three different branching patterns of the main coronary arteries

Coronary artery angiography was performed in 50 normal Lewis rats. The rats had three major coronary arteries; the left coronary artery, right coronary artery and septal artery. The septal artery invariably branched off either from the proximal part of the left coronary artery (n = 30, 60%) or the right coronary artery (n = 20, 40%) (Fig 1D and 1E). It then descended along the right surface of the interventricular septum toward the apex of the left ventricle (LV) (Fig 1D–1F). The left anterior descending artery (LAD) that branched off from the left coronary artery coursed obliquely across the left ventricular free wall towards the apex of the heart and supplies the left ventricular wall by numerous almost superficial lateral branches. The LAD did not branch off into the interventricular septum (septal myocardium), which contrasts with human coronary vessels.

**Fig 2:**
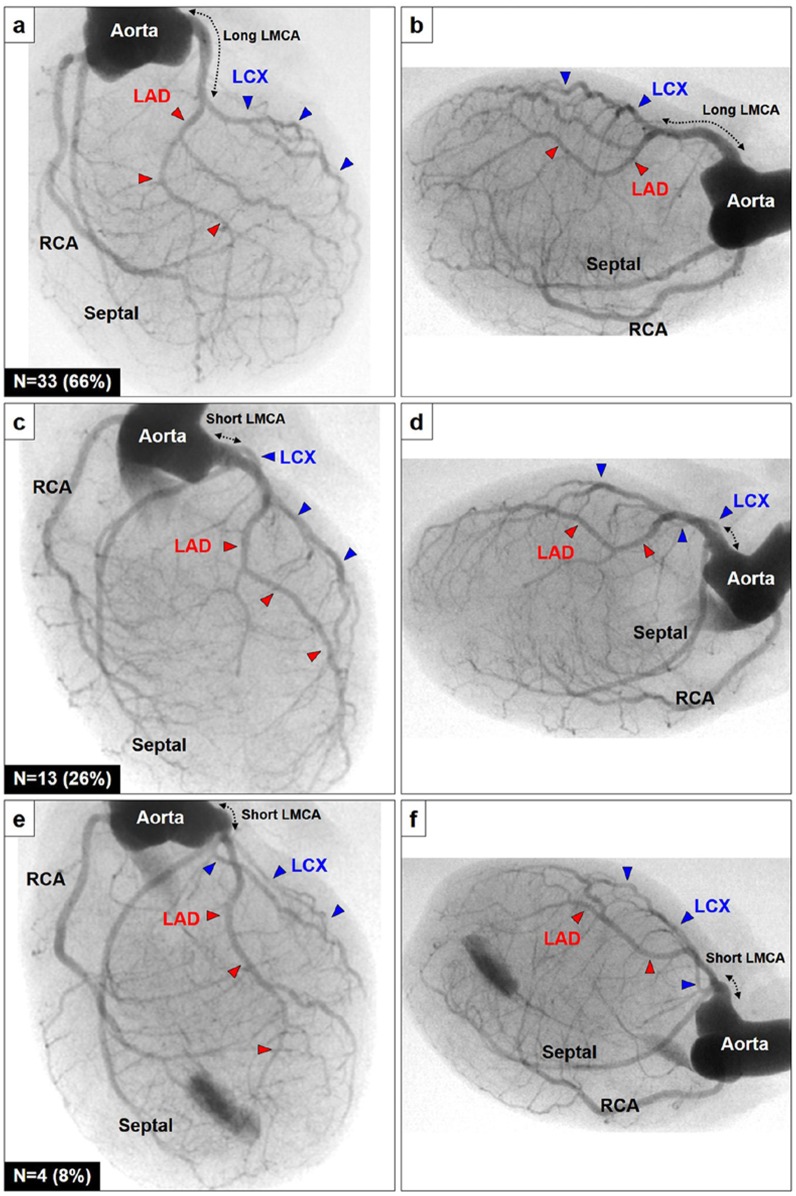
There are 3 branching patterns of LCX, which arose distally from a long LMCA (A, B), proximally from a short LMCA (C, D), or the septal artery (E, F). Abbreviations, see Fig 1, LMCA = left main coronary artery.

In regard to the branching patterns of the left coronary artery, three branching patterns of the left circumflex artery (LCX) were observed: 33 (66%) showed LCX branching off from a relatively long LMCA peripherally (Fig 2A), while the remaining 17 (34%) had a relatively short LMCA and the LCX branched proximally off either from the LMCA (n = 13, 26%) or from the septal artery (n = 4, 8%) (Fig 2B and 2C). Interestingly, all of the 20 rats that displayed the septal artery branching off the proximal part of the right coronary artery had a long LMCA and the LCX branched off peripherally.

### Morphological and angiographical features following ligation of the coronary artery

**Fig 3:**
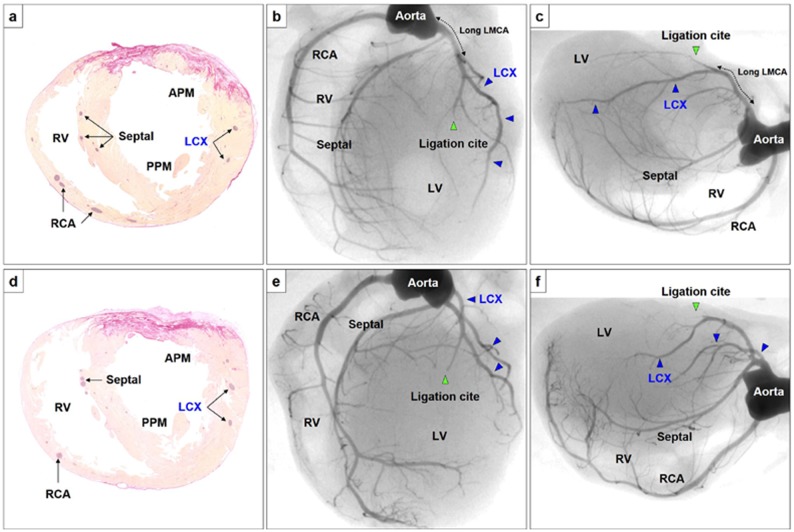
The representative histological and angiographic findings of the 2 rats in which the coronary artery was ligated 2 mm distal to the LAA (A-C and D-F). Both cases showed myocardial infarction localized to anterior territory with intact LCX and septal artery. The size of myocardial infarction was 33% **(A)**, and 26% **(C)**, respectively. Abbreviations, see Figs 1 and 2.

**Fig 4:**
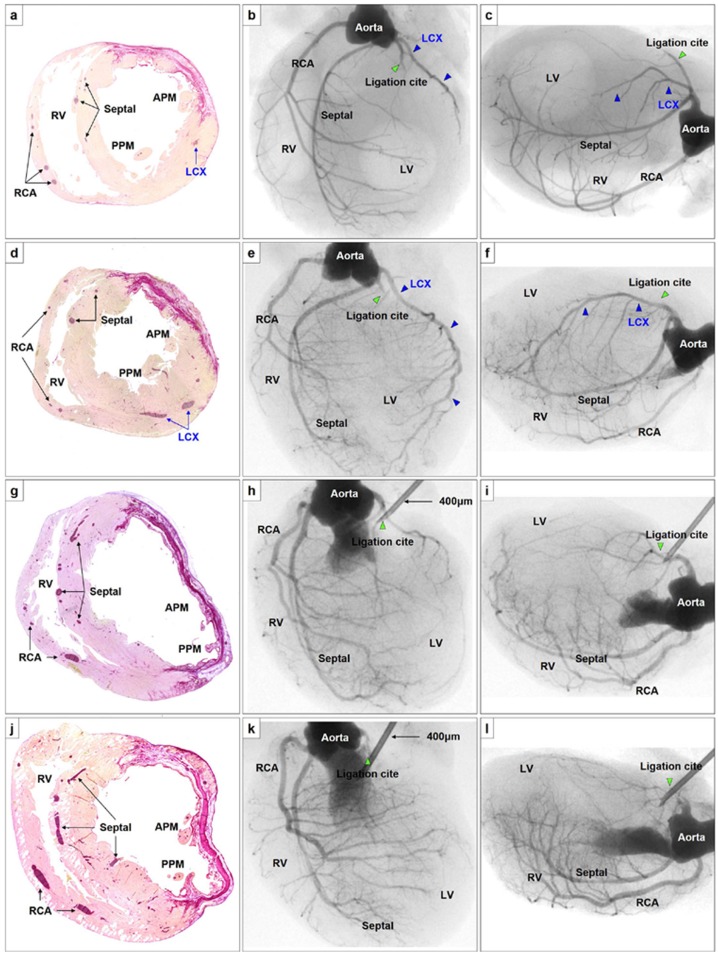
The representative histological and angiographic findings of the 4 rats in which the coronary artery was ligated immediately below the LAA **(A-I)**. The former 2 rats showed myocardial infarction localized to anterior territory with intact LCX **(A-C and D-F)**, while the latter 2 showed broad myocardial infarction involving anterior and lateral territories **(G-I and J-L)**. The size of myocardial infarction was 35% **(A)**, 27% **(D)**, 51% **(G)**, and 53% **(J)**, respectively. The territory of the septal artery was not affected in any of the rat models of myocardial infarction. Abbreviations, see Figs 1 and 2.

In a chronic myocardial infarction model, coronary artery angiography was also performed 3 weeks after coronary ligation immediately below left atrial appendage or 2mm distal to the left atrial appendage (n = 25 for each). In these models, angiography revealed that the coronary artery was without exception ligated distal to the origin of the septal coronary artery, irrespective of the ligation site. The septum was therefore spared from infarction in all of the myocardial infarction rat models (Figs 3 and 4).

In the models in which the coronary artery was ligated 2 mm distal to the left atrial appendage, the histological images showed that the size of myocardial infarction invariably localized to an anterior territory and the LCX was intact (Fig 3A–3F). Corresponding with the histological findings, angiography also showed an intact LCX irrespective of differences both in the length of LMCA and LCX branching pattern. The anterior territory appeared devoid of large arteries, but was accompanied by several small collateral vessels.

In the models in which the coronary artery was ligated immediately below the left atrial appendage, 9 (36%) had a myocardial infarction localized to anterior territory (Fig 4A–4F), while the remaining 16 (64%) had broad myocardial infarction involving anterior and lateral territories (Fig 4G–4L). In the rats that presented with myocardial infarction localized to the anterior territory only, angiography revealed an intact LCX that invariably branched off from a short LMCA. On the other hand, in the rats that presented with broad myocardial infarction, both the LAD and the LCX were found to be ligated and their territories appeared devoid of large arterial vessels.

**Fig 5:**
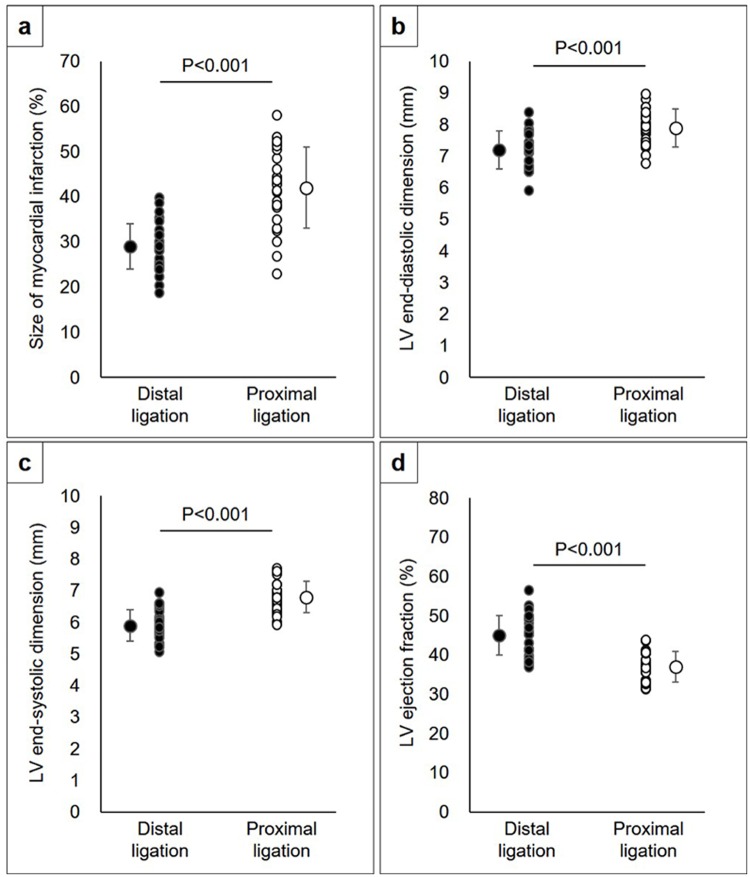
Scatter plot showing size of myocardial infarction (A), LV end-systolic dimension (B), and LV ejection fraction (C) in the models in which the LCA was ligated distally versus proximally.

As a result, the mean size of the myocardial infarction was 42±9% (range, 23–58%) in the proximally ligated models and 29±5% (range,19–40%) in the distally ligated models, respectively (p<0.001) (Fig 5A).

### Impact of coronary ligation on papillary muscle ischemia

In the models with anterior myocardial infarction (n = 34), both anterior and posterior papillary muscles were intact. In contrast, in the model with broad infarction involving anterior/lateral territories (n = 16), the anterior papillary muscle was without exception atrophied and half of them (n = 8) showed myocardial infarction involving the posterior papillary muscle as well. These data indicate that the anterior papillary muscle is not supplied by the LAD, but mainly by the LCX and the posterior papillary muscle might be supplied by the LCX, septal artery or right coronary artery.

### Cardiac function following myocardial infarction

**Fig 6:**
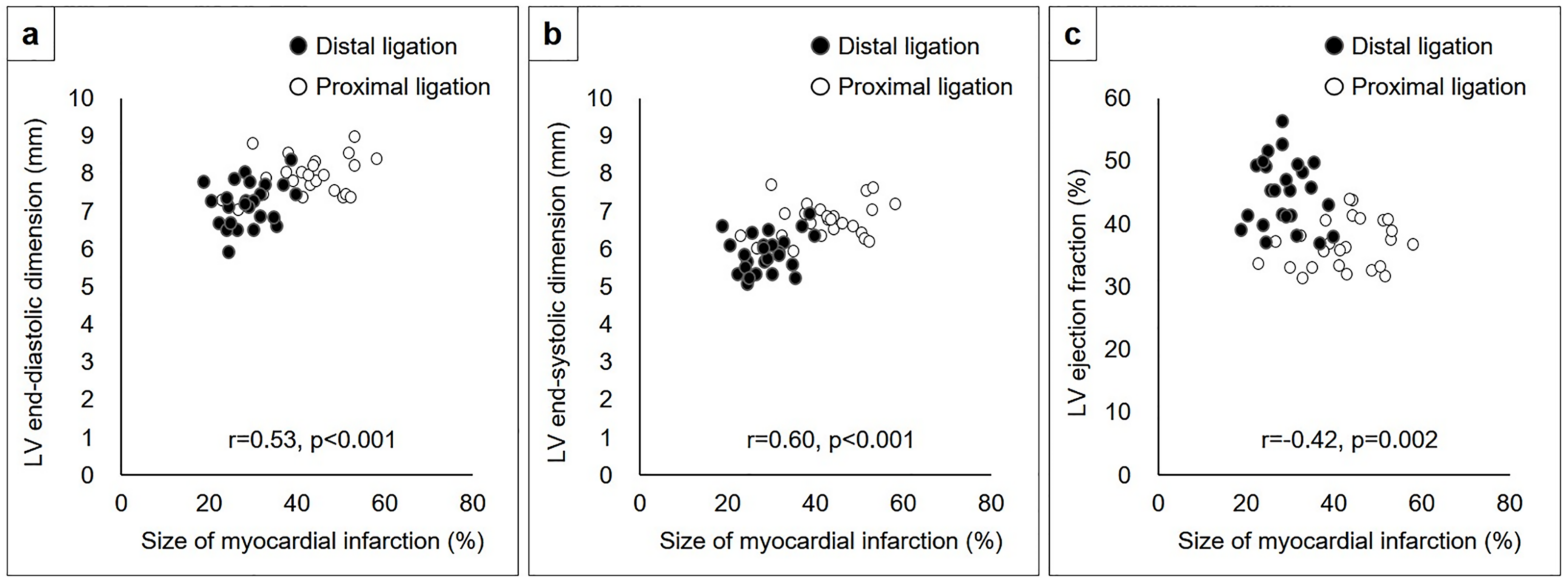
Relationship between the size of myocardial infarction and LV end-diastolic dimension (A), LV end-systolic dimension (B), and LV ejection fraction (C). The black circles indicate the models in which the coronary artery was ligated 2 mm distal to the LAA and the white circles indicate the models in which the LAD was ligated immediately below LAA.

Echocardiography showed mildly impaired LV systolic function with anterior wall thinning (S1 Video) in the models in which the coronary artery was ligated 2 mm distal to the left atrial appendage, whereas it showed moderately to severely impaired LV systolic function with anterior and lateral wall thinning in 64% of the models in which the coronary artery was ligated immediately below the left atrial appendage (S2 Video). Corresponding with the histological findings, there were significant differences in the LV dimensions and systolic function between the rats in which the coronary arteries were ligated distally versus proximally (LV end-diastolic dimension; 7.2±0.6 mm versus 7.9±0.6 mm, LV end-systolic dimension; 5.9±0.5 mm versus 6.8±0.5 mm, LV ejection fraction; 45±5% versus 37±4%, p<0.001 for all) (Fig 5B and 5C). The mean size of the myocardial infarction was positively correlated with LV end-diastolic (r = 0.53, p<0.001) and systolic dimensions (r = 0.60, p<0.001), while negatively correlated with LV ejection fraction (r = -0.42, p = 0.002) (Fig 6A–6C).

## Discussion

The major findings of this study can be summarized as follows; in the female Lewis rat, (1) there were three major coronary arteries including the left coronary artery, right coronary artery and septal artery which branched off either from the proximal part of the left coronary artery or right coronary artery, (2) there were three branching patterns of the LCX: more than 60% showed the LCX branching distally off a relatively long LMCA, while the remainder had a relatively short LMCA and an LCX proximally branching off the LMCA most frequently, and to a lesser extent the septal artery, (3) the size ofmyocardial infarction localized to the anterior territory of the LV in rats in which the coronary artery was ligated 2mm distal to the left atrial appendage, but more widespread to lateral regions when ligated immediately below the left atrial appendage, (4) creation of myocardial infarction involving the septal territory might be impossible because of its anatomical location, and (5) the degree of LV dysfunction after myocardial infarction was closely related to infarct size and location.

### Differences in the coronary architecture between humans and the Lewis rat

In the human, the LAD supplies the territories including anterior wall of LV and the anterior two thirds of the septum. However, Lewis rats did not possess an anatomical LAD tracking down the interventricular septum and ligation of the so-called LAD in rats, as we have demonstrated, consistently caused infarction of the free wall of the LV, while sparing the septum. The angiographic finding that the septal artery invariably originated above the site where the coronary occlusion was performed clearly accounted for this phenomenon. In fact, a large number of the studies that used a rodent LAD-ligation model have consistently shown the intact interventricular septum [14–17], which would be explained by the anatomical findings of this study. It may be technically possible to ligate the septal artery at its origin near the aortic root, however the mortality rate is considered to be extremely high. Since the LAD perfuses a different region in the rat to that of the human, it does not perform the same function as in humans. Taken together, the distinct rat coronary artery anatomy resulted in different territories of myocardial infarction compared with human and large animals, and therefore affected the associated pathological post-infarct ventricular remodeling.

### Influence of left coronary architecture on the variability in myocardial infarction

Our data clearly showed that different size and locations of myocardial infarction can be created by the conventional coronary ligation technique in Lewis rats, based on differences in the site of coronary ligation and left coronary artery anatomy. The angiography findings imply that it would be difficult to ligate the LCX which branched off proximally from a short LMCA even if the coronary artery is ligated immediately below left atrial appendage. The proportion of normal rats that had the LCX branching off from the short LMCA (34%) was almost comparable with the proportion of myocardial infarction rat models localized to the anterior territory (36%), indicating that the different size of myocardial infarction created was at least partly attributed to the LCX branching patterns. Our data also leads us to consider that coronary artery should not be ligated immediately below left atrial appendage, but rather 2 mm distal to the left atrial appendage when aiming to create myocardial infarction model localized to an anterior territory. Conversely, when targeting a broad (e.g. anterior plus lateral territories) myocardial infarction model mimicking patients with chronic heart failure and advanced ischemic cardiomyopathy, the coronary artery should be ligated immediately below the left atrial appendage. Most importantly, we should be aware that even when the left coronary artery is ligated immediately below left atrial appendage in a similar way by well-trained researchers, there is a risk that approximately one-third of the rat model will develop a relatively small myocardial infarction localized to the anterior wall and reduced impairment of LV systolic function than expected. In addition, to ensure that only animals with uniform infarcts are being compared, investigators should classify their animals into groups with different infarct sizes at the time of randomization, and thus a larger number of animals would be required to achieve a maximum size of infarction.

### Differences in the coronary architecture in the rodents based on sex

Previous studies which investigated coronary morphology in the rodents mostly focused on males, for example [5, 8, 10], and we are not aware of any studies that identify differences between sexes. In a previous investigation by Liu YH and colleagues, 83% of the male Lewis rats (5 out of 6) had left LCX branching off from LMCA proximally to the edge of left atrial appendage, while the septal artery branched off either from the left or right coronary artery near the aortic root, which was quite consistent with the coronary morphology in the female Lewis rat shown in our study. In addition, in our labs we have performed angiography and micro CT on mice and rats of both sexes and have found no evidence at all to suggest that the variability in coronary branching patterns is linked to sex.

It is important, however, to understand coronary anatomy and the varying degrees of myocardial infarction induced by coronary artery ligation technique in female rodents, as they are often used as a recipient in cell based therapy [18–21]; as real-time polymerase chain reaction for the Y-chromosome-specific sry gene is useful for quantifying the presence and retention of transplanted donor cells, which has been shown to be closely related to therapeutic effects many times, in a model in which the donor (male) cells were transplanted in the damaged female heart. Taken together, we believe that our data are useful for other investigators in interpreting the numerous studies carried out in this animal model.

## Limitations

In this study, we focused on the coronary anatomy and its relation to the varying size and location of myocardial infarction model created by coronary artery ligation in female Lewis rats that are routinely used in studies of regenerative therapies [22]. Therefore, the application of our data to other strains of rats should be taken with caution.

## Conclusions

Infarct size variability was caused not only by ligation site but also by varying LCX branching patterns. There are potential risks of creating different size and locations of myocardial infarction, particularly when targeting a broad range of myocardial infarction. The territory of the septal artery always appears to be spared from myocardial infarction induced by the coronary ligation technique.

## Supporting information

S1 Video Echocardiography indicating mildly impaired LV systolic function with anterior wall thinning. (MP4)

S2 Video Echocardiography indicating moderately to severely impaired LV systolic function with anterior and lateral wall thinning. (MP4)
